# Supplementary figures and images for: Genetic Diversity and Genome-Wide Association Study for the Phenology Response of Winter Wheats of North America, Western Asia, and Europe
Source: Plants (Basel). 2023 Dec 1;12(23):4053. doi: 10.3390/plants12234053 (PMC10708061; doi:10.3390/plants12234053)

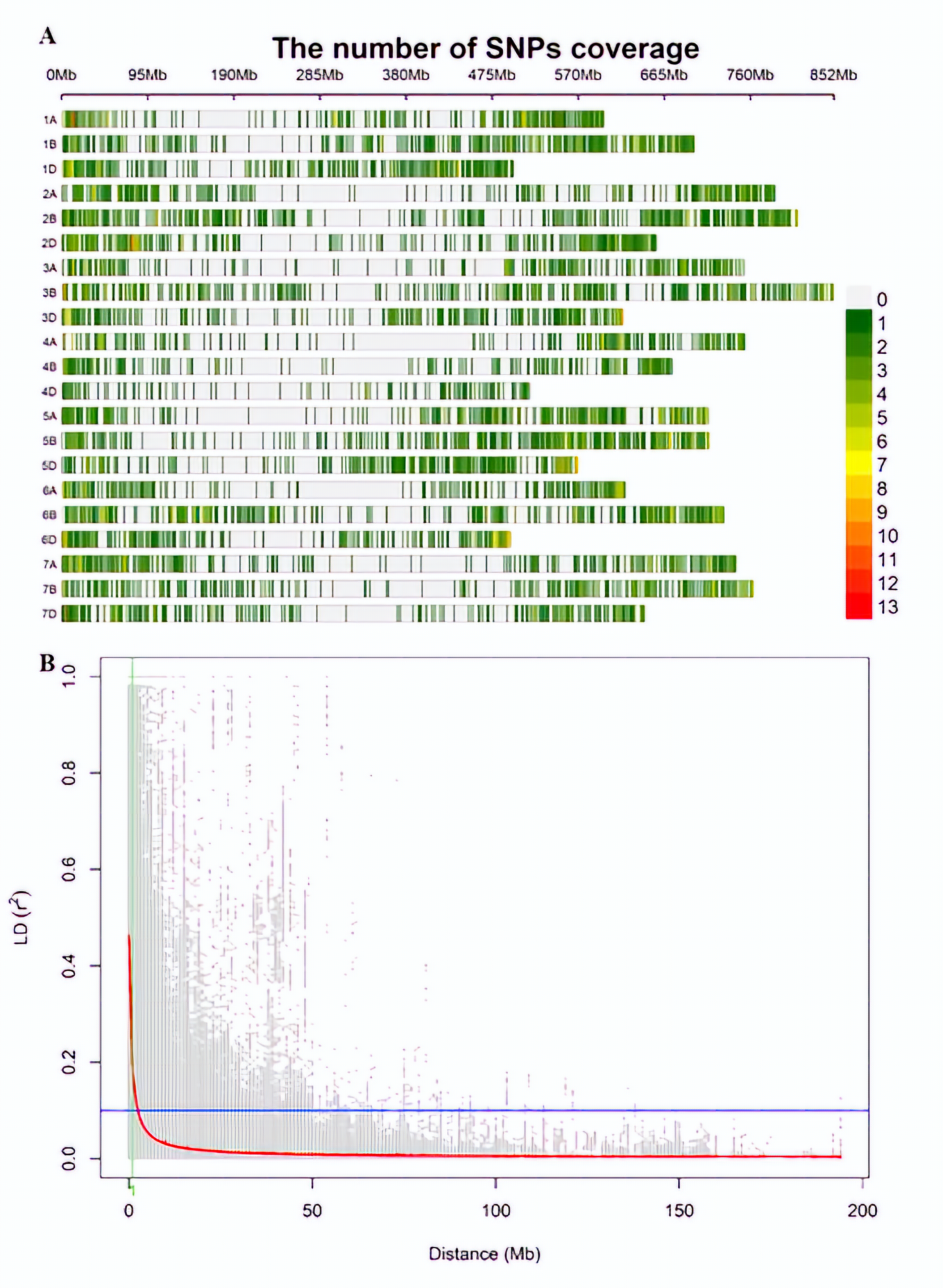

Supplement: Supplementary file 1 [file plants-12-04053-s001.zip › Figure. S1.png]
